# QuickStats

**Published:** 2013-01-11

**Authors:** Samara Joy Nielsen

**Figure:**
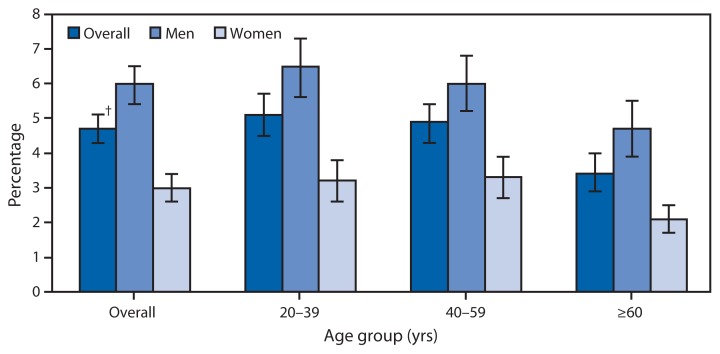
Average Percentage of Daily Calories from Alcoholic Beverages^*^ Among Adults Aged ≥20 Years, by Age Group — National Health and Nutrition Examination Survey, United States, 2007–2010 ^*^ Alcoholic beverages include beer, wine, liquor, and mixed drinks (cocktails). Data on consumption are based on in-person, 24-hour dietary recall interviews. ^†^ 95% confidence interval.

During 2007–2010, on average, 4.7% of the daily calories consumed by U.S. adults aged ≥20 years came from alcoholic beverages. The percentage of daily calories from alcohol ranged from 6.5% for men aged 20–39 years to 2.1% for women aged ≥60 years. Across age groups, the percentage of calories from alcohol was higher among men; among both men and women, the percentage declined with age.

**Sources:** National Health and Nutrition Examination Survey, 2007–2010. Available at http://www.cdc.gov/nchs/nhanes.htm.

Nielsen SJ, Kit BK, Fakhouri T, Ogden CL. Calories consumed from alcoholic beverages by U.S. adults, 2007–2010. NCHS data brief, no. 110. Hyattsville, MD: US Department of Health and Human Services, CDC, National Center for Health Statistics; 2012. Available at http://www.cdc.gov/nchs/data/databriefs/db110.htm.

